# Supplementary figures and images for: A data-driven approach for examining the demand for relaxation games on Steam during the COVID-19 pandemic
Source: PLoS One. 2021 Dec 16;16(12):e0261328. doi: 10.1371/journal.pone.0261328 (PMC8675663; doi:10.1371/journal.pone.0261328)

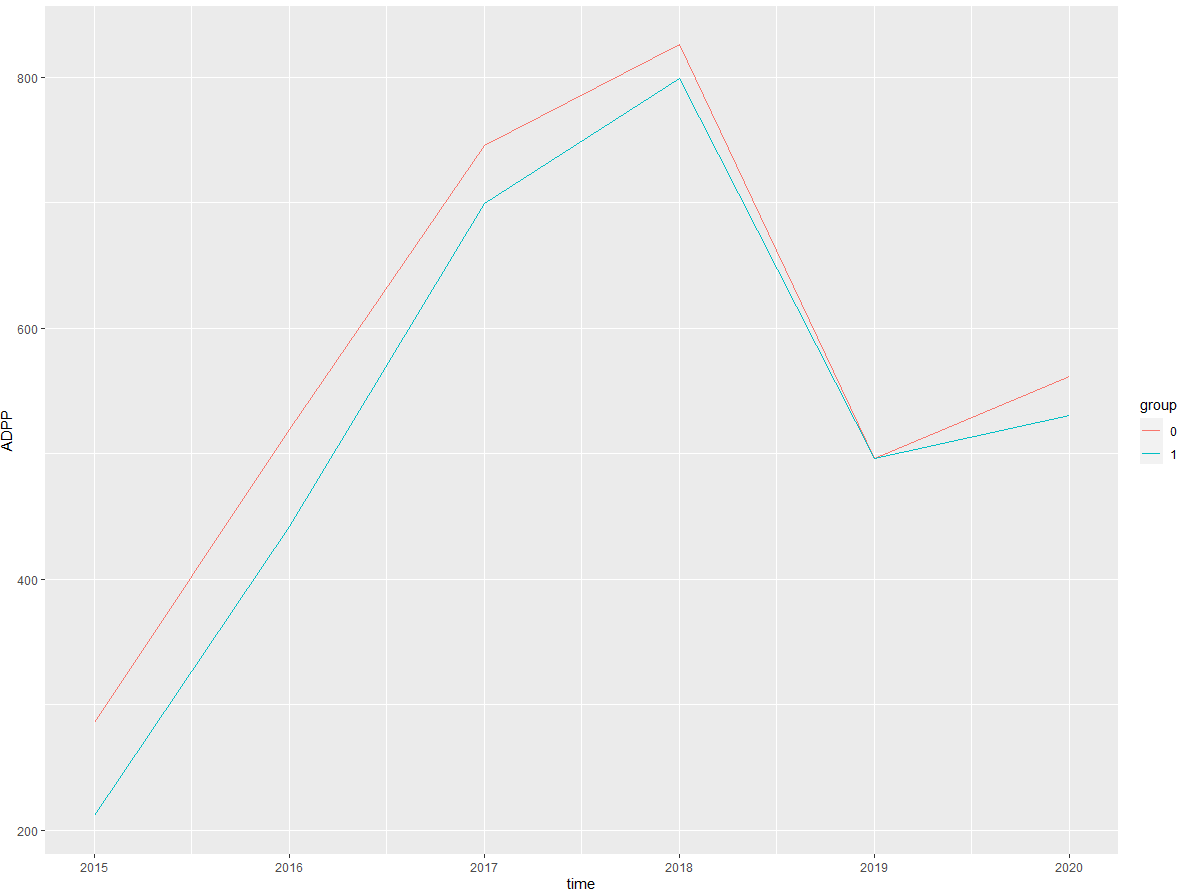

Supplement: S1 Fig — (TIF) [file pone.0261328.s001.tif]
